# Do intensivists prognosticate patients differently from themselves or their loved ones?

**DOI:** 10.1186/cc14649

**Published:** 2015-03-16

**Authors:** S Gupta, C Green, R Tiruvoipati, J Botha

**Affiliations:** 1 Peninsula Health, Frankston, Australia

## Introduction

There is a paucity of data about whether our treatment philosophy is different for our patients as compared with what we would have wanted for ourselves, or while acting as surrogate decision-makers for our loved ones.

## Methods

An anonymous survey was sent to all the members of Australia and New Zealand Intensive Care Society and the College of Intensive Care Medicine (CICM). The first section comprised a hypothetical case scenario spanning over 6 weeks of ICU stay for a patient. At four different stages of the ICU stay, responders were requested to answer multiple-choice questions regarding the philosophy of treatment, based on their perceived prognosis of the patient at that particular time. The following two sections contained the same set of questions with the hypothetical scenario of responders acting as surrogate decision-makers for the patient and that of responders being patients themselves, in the same situation. The responses were compared amongst three sections at each stage using the chi-square test.

## Results

A total of 115 responses were received from the fellows of CICM. The results are presented in Tables 1 and 2.

**Table 1:** Respondents advocating withdrawal for the patient.

	Withdrawal self (%)	Withdrawal family (%)
Day 3	71	67
Day 7	83	76
Day 28	96	88
Day 42	98	97

**Table 2:** Respondents advocating continuing care for the patient.

	Withdrawal self (%)	Withdrawal family (%)
Day 3	16	10
Day 7	23	15
Day 14	25	19
Day 42	42	29

## Conclusion

Of the ICU physicians who would withdraw care for their patient, the majority would also want the same for themselves. The disparity between decision to continue to treat the patients versus treating self or family increased with increasing length of stay.

## References

[B1] Korones DN What would you do if it were your kid? N Engl J Med 2013 369 1291 3 10.1056/NEJMp1304941 24088090

